# Correlation nitric oxide level and homeostatic model assessment insulin resistance in obese adolescent

**DOI:** 10.1186/1687-9856-2013-S1-P84

**Published:** 2013-10-03

**Authors:** Julinar, Eka Agustia Rini, Hafni Bachtiar, Fadil Oenzil

**Affiliations:** 1 Endocrinology Division, Pediatric Health Departement of Medical Faculty Andalas University, Dr.M.DjamilHospital Padang

**Keywords:** Obesity, nitrit oxide, HOMA-IR, insulin resistance

## Background

childhood obesity is a significant health problem that has reached epidemic proportions around the world. Childhood obesity associated with increased risk for several cardiovascular and metabolic syndrome, such as insulin resistance. Homeostatic model assessment insulin resistance (HOMA-IR) is a marker widely used for insulin resistance. Nitrit oxide (NO) has important role in insulin resistance.

## Objective

To determine correlation between NO and HOMA-IR in obese adolescent.

## Method

A cross sectional study was performed in 44 obese senior hight school students in Padang from December 2011 until March 2012. Chosen through multi stage random sampling, devidedinto 2 groups; 22 in insulin resistance and 22 in non insulin resistance. Variables measured were HOMA-IR with calculated based on fasting glucose and insulin level, serum NO levels. Data was analyzed statistically with computerization system using regression correlation test and T-test. P<0.05 was significant.

## Result

There was significant difference in body mass index between insulin resistance and non resistance group (32.53+2.57 vs 30.55+3.03 kg/m^2^). Glucose level was no significant difference between insulin resistance and non resistance group (4.93+0.79 vs 4.66+1.02 mmol/L). insulin was significant difference between insulin resistance and non resistance group (20.23+4.03 vs 15.82+5.72 uIU/ml. Nitrit oxide level was significant difference between insulin resistance and non resistance group (70.07+24.98 vs 55.04+19.66 umol/ L. There was significant correlation between NO level and HOMA IR (r=0.482; p=0.001) and no significant correlation between BMI and NO levels (r=0.135;p=0.325).

## Conclusion

Nitrit oxide is significantly associated with HOMA-IR in obese adolescent.

